# Sex-dependent effects on tasks assessing reinforcement learning and interference inhibition

**DOI:** 10.3389/fpsyg.2015.01044

**Published:** 2015-07-22

**Authors:** Kelly L. Evans, Elizabeth Hampson

**Affiliations:** Department of Psychology, University of Western Ontario, LondonON, Canada

**Keywords:** sex difference, reversal learning, reward processing, inhibitory control, prefrontal cortex

## Abstract

Increasing evidence suggests that the prefrontal cortex (PFC) is influenced by sex steroids and that some cognitive functions dependent on the PFC may be sexually differentiated in humans. Past work has identified a male advantage on certain complex reinforcement learning tasks, but it is unclear which latent task components are important to elicit the sex difference. The objective of the current study was to investigate whether there are sex differences on measures of response inhibition and valenced feedback processing, elements that are shared by previously studied reinforcement learning tasks. Healthy young adults (90 males, 86 females) matched in general intelligence completed the Probabilistic Selection Task (PST), a Simon task, and the Stop-Signal task. On the PST, females were more accurate than males in learning from positive (but not negative) feedback. On the Simon task, males were faster than females, especially in the face of incongruent stimuli. No sex difference was observed in Stop-Signal reaction time. The current findings provide preliminary support for a sex difference in the processing of valenced feedback and in interference inhibition.

## Introduction

Emerging evidence suggests the PFC may be a sexually differentiated brain region in humans and may be responsive to sex steroids. Sex steroids exert two classes of effects in the brain. Permanent effects on neural structure in responsive regions of the nervous system take place during prenatal or perinatal development and are referred to as *organizational effects. Activational effects* are a result of hormones currently in the bloodstream of adults and are reversible, often entailing alterations in neurochemistry. Sex differences can be a product of organizational effects, activational effects, or a combination of the two (Breedlove and Hampson, 2002). Although the localization of hormone receptors sometimes differs between the two sexes (e.g., Sholl and Kim, 1990), brain differences caused by steroids more commonly reflect the large difference in ligand availability between the two sexes. Androgen receptor-immunoreactivity in the OFC of developing and adult male and female rhesus monkeys (Clark et al., 1988; Finley and Kritzer, 1999) suggests that androgens can act in the primate OFC. Recent evidence has revealed that androgens increase spine synapse density in the PFC of adult vervet monkeys (Hajszan et al., 2008) and can modulate neurotransmitter systems of the PFC including the dopamine and serotonin systems (Handa et al., 1997; Aubele and Kritzer, 2011).

Consistent with the possibility that the PFC is responsive to sex steroids, there are reports of sex differences at the functional level (e.g., Duff and Hampson, 2001; Overman, 2004; van den Bos et al., 2013). On average, adult males learn the deck contingencies of the IGT more rapidly than do females (e.g., Reavis and Overman, 2001; Bolla et al., 2004; Weller et al., 2009). Sex differences in brain activation have been found during IGT performance with males showing increased activation in the right and left lateral OFC and females showing increased activation in the left medial OFC (Bolla et al., 2004). Similarly, a male advantage has been found on an object reversal task dependent on the OFC in infant monkeys (Goldman et al., 1974; Clark and Goldman-Rakic, 1989) and young children (15 to 30 months of age; Overman et al., 1996), and on a probabilistic reversal learning task in adults (Evans and Hampson, 2015; but see Overman, 2004). However, the IGT and reversal learning are both complex tasks that involve multiple component processes and the functional task element that leads to the male advantage has not been identified (see Overman, 2004 and van den Bos et al., 2013 for reviews). Prominent task elements include inhibitory control and learning based on reward and/or punishment. A sex difference in one or more underlying processes could give rise to the male advantage observed.

Inhibitory control is an important component of performance on both tasks. On the IGT, participants are initially drawn to the “bad” decks in which the reward payout is higher, but in order to optimize performance they must learn to inhibit this attraction as these decks lead to monetary losses over time. Participants must also inhibit the tendency to shift their choices from the “good” decks to the “bad” decks upon encountering a loss in a “good” deck. Likewise, during reversal learning, participants must learn, after a reversal takes place, to inhibit their responses to the stimuli that were rewarded during acquisition. Empirical evidence is mixed regarding whether a sex difference might exist in response inhibition. Response inhibition has been hypothesized to comprise several subtypes such as action cancelation, interference inhibition, and action withholding (Sebastian et al., 2013). With respect to action cancelation tasks, several reports have failed to find a sex difference in SSRT (Williams et al., 1999; Li et al., 2006, 2009; Cross et al., 2011). For action withholding tasks such as the go/no-go paradigm, females have been found to be better at inhibiting a response than males in some studies (Hooper et al., 2004; Hansen, 2011), but not others (Garavan et al., 2006; Cross et al., 2011; Liu et al., 2012). For tasks that involve interference inhibition, no sex differences have been found on the Stroop (MacLeod, 1991; Cross et al., 2011; Veroude et al., 2013), but some reports suggest a male advantage on tasks that involve inhibiting responses to obvious stimuli in favor of less obvious stimuli (Halari and Kumari, 2005; Halari et al., 2005) or on other types of interference inhibition tasks (Stoet, 2010; Clayson et al., 2011). Thus, at present, the literature is inconclusive with respect to sex differences and it is unclear whether the inconsistencies are due to differences in the types of inhibition examined.

Both the IGT and reversal learning involve receiving reward and punishments, either in the form of winning/losing ‘virtual’ money, points, or in animal studies, food reward. There is evidence that the affective value of both primary and abstract secondary reinforcers (including social approval/disapproval) is represented in the OFC (Elliott et al., 1997; Kringelbach and Rolls, 2004). Previous work finding a sex difference on the IGT is consistent with the possibility that the reward and punishment element of the task is important as females tend to select more cards than males from the deck with large, frequent reward and a low frequency of punishments (e.g., Overman, 2004; Evans and Hampson, 2015). All reversal tasks used experimentally are based on the provision of reward or punishments, contingent upon the responses that are made, and prompt utilization of feedback becomes especially important upon reversal when respondents must switch their choice to the other object in the pair. Thus, it might be the case that males and females differ in their use of, or sensitivity to, reward and punishment information. Support for the idea that reward and/or punishment processing may be a key component comes from several studies. On a simple decision-making task, Weller et al. (2009) found that while there was no sex difference in making risky choices related to potential gains, women took more risks than men when it came to potential losses. Robinson et al. (2010) found a sex difference in punishment-related reversal learning after a procedure to reduce global dopamine synthesis, whereby females displayed improved reversal learning based on punishment after dopamine depletion. However, reward-related reversal learning was unaffected. A further study found that women activated the medial PFC at the time of reward delivery more strongly than men during a slot machine task that varied reward probability, magnitude, and expected value (Dreher et al., 2007). Finally, there is evidence that females discount hypothetical reward more so than males during delay discounting tasks (see Hosseini-Kamkar and Morton, 2014; Weafer and de Wit, 2014 for reviews). Thus, some limited evidence supports the possibility of sex differences when learning from reward and punishment. If this is true, it potentially could be a functional component leading to the observed male advantage on the IGT and reversal learning tasks.

The objective of the current study was to begin to illuminate which task components are the key source of the male advantage. To test whether there is a sex difference in response inhibition, we used two inhibitory control tasks. The Stop-Signal Task (Cambridge Cognition) assessed action cancelation and the Arrows Task (Davidson et al., 2006) assessed interference inhibition. The Probabilistic Selection Task (PST; modified from Frank et al., 2004) was used to test for sex differences in learning from positive and negative feedback in the absence of reversal. If the male advantage on the IGT and reversal learning tasks stems from a sex difference in the processing of positive and/or negative feedback, then a sex difference in performance on the PST would be predicted.

## Materials and Methods

### Participants

Healthy young participants were recruited from the University of Western Ontario and received monetary compensation or course credits for participating. Only participants with no history of neurological (e.g., sports-related head injury) or mental health conditions, and not on psychoactive medications or oral contraceptives were considered eligible. Oral contraceptives suppress the production of ovarian hormones including androgens and thus have a potential to alter reward processing and/or response inhibition (Amin et al., 2006; Smith et al., 2014). On a mood questionnaire administered as part of the testing, nine participants nonetheless showed evidence of active depression and had to be excluded. Because several of our tasks involved complex instructions and adequate comprehension was necessary to ensure the validity of the resulting test scores, any participant with English as a second language who scored more than 1.0 SD below the mean (based on local test norms) on the Verbal Meaning Test (a test of vocabulary knowledge administered during the test session; Thurstone and Thurstone, 1963) was not included in statistical analyses (*n* = 12). There were 176 participants in the resulting sample (90 males, 86 females) with a mean age of 19.96 for males (range = 17–30 years) and 20.31 for females (range = 17–31 years). Participants provided written informed consent before taking part in the study. The study received ethical approval from the Research Ethics Board for Non-Medical Research Involving Human Subjects at the University of Western Ontario.

### Experimental Tasks

#### Probabilistic Selection Task (PST; modified from Frank et al., 2004)

**FIGURE 1:**
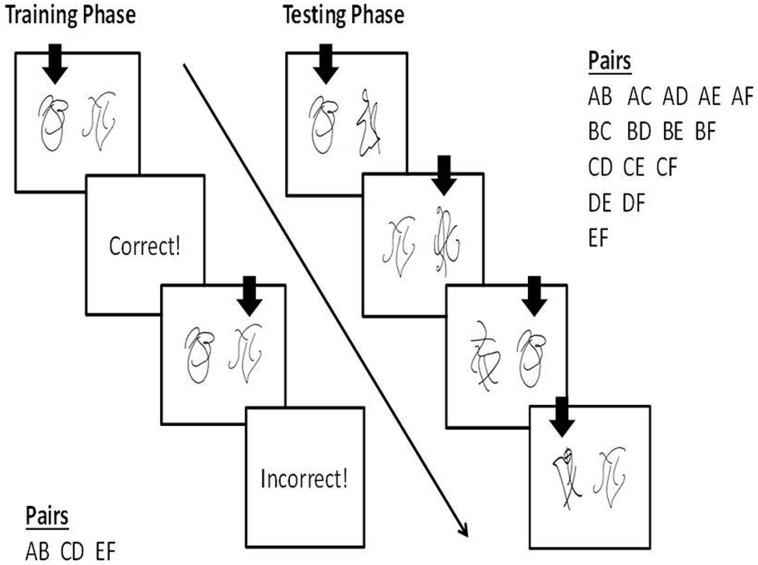
**Probabilistic Selection Task.** The vertical arrows represent hypothetical choices made by a participant. During training, participants had to learn which object in each of three pairs (AB, CD, EF) was correct. The reinforcement contingencies were 85–15 (AB), 75–25 (CD), and 65–35 (EF). After reaching the learning criterion or completing 480 trials, the test phase began. During the test phase, all possible pairings of the six objects were presented. Participants had to select the object they thought was correct in each pair shown, based on their experience from the training phase, without receiving feedback. Learning based on *positive feedback* was measured by the number of times object A was selected in all pairings other than AB. Learning based on *negative feedback* was measured by the number of times object B was avoided in all pairings other than AB.

The PST is a well-established reinforcement learning task that is impaired by damage to ventromedial or orbital PFC (Wheeler and Fellows, 2008). It consists of a training phase and a test phase administered on a computer. During the training phase, participants viewed three pairs of objects one at a time and had to learn which object in each pair was ‘correct’ (**Figure 1**). The objects were abstract line drawings from the Self-Ordered Pointing task of Petrides and Milner (1982). The participant selected one of the objects from each pair by pressing one of two buttons on a response box and verbal feedback was provided (“*Correct!*” printed in blue or “*Incorrect!*” printed in red). The feedback was probabilistic and the reinforcement contingencies differed for each pair. The first pair (Pair AB) was 85–15 (object A was ‘correct’ on 85% of trials, object B was ‘correct’ on 15% of trials), the second pair (Pair CD) was 75–25, and the third pair (Pair EF) was 65–35. The pairs were presented in blocks of 60 trials (20 trials of each pair). The participants continued in the training phase until they reached a designated learning criterion or until 480 trials were completed. The learning criterion was choosing A over B in 70% of trials within a block as has been used in past research by other labs (Wheeler and Fellows, 2008; Rustemeier et al., 2012). Some studies have adopted slightly different criteria for learning (e.g., 65% A in AB, 60% C in CD, and 50% E in EF; Frank et al., 2005). However, we chose to use the AB criterion only, because learning to prefer A over B is the only prerequisite for successful performance during the test phase (Rustemeier et al., 2012).

Because both A and B were always presented together during the training phase, a participant potentially could reach criterion by learning to choose A, avoid B, or a combination of the two. Thus, to dissociate these two different types of learning, the objects were recombined to form all possible combinations during the test phase (including the original three pairings) and the participants performed the same task, this time without receiving feedback. Each pair was presented three times (90 trials in total). The number of trials in which the object reinforced the most during training (i.e., object A reinforced 85% of the time) was chosen in the novel pairs (AC, AD, AE, AF) represented a measure of learning from positive feedback. The number of trials in which object B, the object reinforced the least during training (15% of the time), was avoided in the novel pairs (BC, BD, BE, BF) represented a measure of learning from negative feedback.

Twelve matched versions of the PST were created to ensure that all objects had a chance to be object A and object B (e.g., object A = item 1 and object B = item 2 in one version) and that every object was the reinforced object in the AB pair (e.g., object A = item 2 and object B = item 1 in another version). Stimuli were presented using E-Prime 2.0.

#### Arrows Task (Davidson et al., 2006)

The Arrows task was used to assess interference inhibition. On each trial, a single arrow was presented on the left or right side of the computer screen. The participant was told to press the left or right button on a response box located in front of the screen, depending on where the arrow was pointing. On congruent trials, the arrow pointed straight down toward the left or right button (arrow and button press on the same side). On incongruent trials, the arrow appeared on the left or right of the screen but pointed diagonally toward the contralateral button (right or left button, respectively, i.e., arrow and button press on opposite sides). The time required to respond (*Speed*, calculated as the median reaction time in milliseconds based on trials in which a correct button press was made) and the number of correct responses (*Accuracy*, the percentage of correct responses) were computed separately for congruent and incongruent trials. Any response time less than 200 ms was considered too fast to be made in response to an arrow and thus was considered to be anticipatory (Davidson et al., 2006). Anticipatory responses were not included when calculating Accuracy or Speed.

The Arrows task is based on the classic Simon paradigm where a specific stimulus (e.g., a picture) is tied to a response on a particular side. Responses are typically more accurate and/or faster when the stimulus and side of the response are congruent than when they are incongruent (the Simon Effect; e.g., Simon and Rudell, 1967; Simon and Berbaum, 1990). The Arrows task was used to provide a measure of interference inhibition as it involved a conflict between responses that were involuntarily co-activated due to incompatible stimulus dimensions (Sebastian et al., 2013). Participants had to inhibit the prepotent tendency to respond on the same side as the arrow on incongruent trials and instead press the button on the side opposite the arrow. The memory load was reduced compared to a Simon task using pictures as the arrow always pointed directly to the correct response. Given the high accuracy rates typically found on this task in past work involving neurologically intact samples (Davidson et al., 2006), it was hypothesized that any observable sex difference would be found on the Speed variable.

#### Stop-Signal Task (Cambridge Neuropsychological Test Automated Battery; CANTABeclipse, Cambridge Cognition Ltd., UK)

On each trial, an arrow was presented inside a fixation circle on the computer screen, pointing horizontally to the left or right. The participant was asked to monitor the direction of the arrow and to press the corresponding button on the response box, using the index finger of the left or right hand, as quickly as possible unless they heard a beep. During a trial with a beep (which occurred on 25% of trials), the participant refrained from responding to the best of their ability.

**FIGURE 2:**
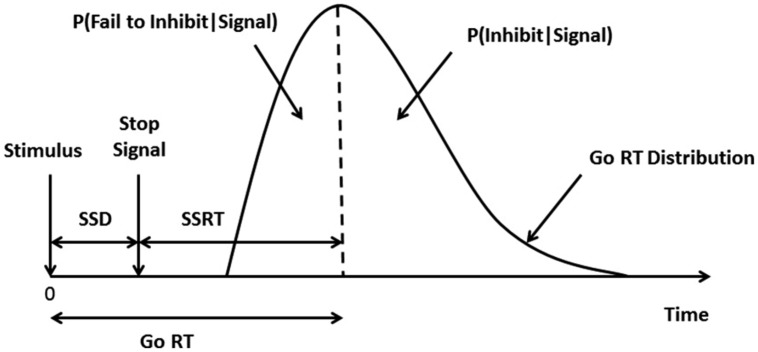
**The Stop-Signal Task.** The theoretical model used to compute the SSRT is called the “horse-race” model. The model assumes that under conditions where a stop signal is given the “stop” and “go” processes race against each other and the process that wins the race will dictate which behavior will be exhibited. The graph depicts the hypothetical distribution of an individual’s go reaction times [median go reaction time (RT) falls at the dotted line]. In trials where a stop-signal is given, the time from the ‘go’ stimulus presentation to the stop-signal presentation is called the SSD. A tracking procedure built into the CANTAB software monitors outcomes and adjusts each individual’s SSD so that the probability of inhibition (P(Inhibit| Signal)) and the probability of responding (P(Respond|Signal)) are equal (i.e., both approximately 50%). This ensures that the SSRT is not biased as the estimate is based on the densest part of the curve (i.e., at 50%), not the tails of the distribution (Band et al., 2003; Leotti and Wager, 2010). To calculate the SSRT, the SSD is subtracted from the median go RT.

Because the behavior of interest in the task is actually the lack of overt behavior (i.e., inhibiting a response), SSRT must be estimated based on a theoretical model. The model commonly used is the “horse-race” model which assumes that there are two processes (the “stop” and “go” processes) that race against one another and the final behavioral outcome depends on which of the two processes wins the race (Logan and Cowan, 1984; **Figure 2**). Only the ‘go’ reaction time, the time to a correct button press in response to a ‘go’ signal (the onset of the arrow), can be computed directly. An indirect method is used to compute the SSRT. Using a tracking procedure built into the software, the delay interposed before the stop-signal occurs SSD is adjusted for each individual such that the timing of the auditory signal will result in successful response inhibition on 50% of trials. The stop-signal is generated based on each participant’s actual performance so that the signal will come later after a successful inhibition trial (making performance on the next stop trial more difficult) and earlier after an unsuccessful inhibition trial (making inhibition on the next stop trial easier) and ensures task difficulty is controlled across participants (Congdon et al., 2012). An individual’s SSD can also be thought of as the amount of handicapping necessary to tie the race between the stop and the go processes (Logan et al., 1997). If one assumes that the stop and go processes finish at the same point in time, this allows for the computation of the SSRT by taking an individual’s SSD and subtracting it from his or her median go reaction time observed over a series of trials.

The Stop-Signal Task was used as a measure of inhibitory control and assesses the ability to cancel an already ongoing motor response (Sebastian et al., 2013). Median go reaction time was calculated for the whole task, and the proportion of successful stops, SSD, and SSRT (in milliseconds) were generated for the last half of the task.

### Control Tasks

#### Verbal Meaning Test (Thurstone and Thurstone, 1963)

This test assessing vocabulary knowledge has 60 items. The participant was allowed 4 min to complete as many items as possible. For each item, the participant chose the word from a list of five alternatives that best matched the meaning of a target word. The score was the number correct. This task was included to assure the groups were matched in overall ability, as vocabulary tasks have been shown to be predictive of general intelligence (Vernon, 1971; Ziegler and Doehrman, 1979; Wechsler, 1981).

#### Positive and Negative Affect Schedule (PANAS; Watson et al., 1988)

The PANAS consists of 20 adjectives that describe different emotions. There are 10 positive adjectives (e.g., interested, excited) and 10 negative adjectives (e.g., distressed, upset). Participants were asked to rate each adjective on a scale that ranged from 1 (*very slightly or not at all*) to 5 (*extremely*), according to how much they felt that way on the day of testing. Total scores were calculated for positive affect and negative affect separately. The PANAS was given at the beginning of the test session, before any cognitive tasks were performed.

### Statistical Analyses

All analyses were done using IBM SPSS 19.0 statistical software. Multivariate analysis of variance (MANOVA) was used to test for a sex difference on each experimental task. The Greenhouse–Geisser epsilon was used to correct for any sphericity violations in the repeated measures variables or interactions (Kirk, 1995). An alpha level of 0.05 was used as the criterion for significance in all comparisons in view of the exploratory nature of the present study.

## Results

### Experimental Tasks

#### Probabilistic Selection Task

##### Training phase

The number of participants who reached the learning criterion in the training phase of the PST was 138 (78% of all participants), consistent with prior studies that used the same criterion used here (e.g., 75%; Rustemeier et al., 2012). Only participants who successfully met criterion were used to analyze the test phase of the PST. The same subset of participants was analyzed for all other tasks in the current study, to allow direct comparisons to be made across the tasks. There was no significant difference between the number of males (68 of 75) and females (70 of 86) reaching criterion, χ^2^(1) = 0.89, *p* = 0.347 nor was there any difference between males (*M* = 184.60, SD = 132.61) and females (*M* = 216.97, SD = 138.64) in the number of trials needed to reach criterion, *F*(1,136) = 1.96, *p* = 0.164, $\eta_{p}^{2}$ = 0.014.

##### Test phase

**FIGURE 3:**
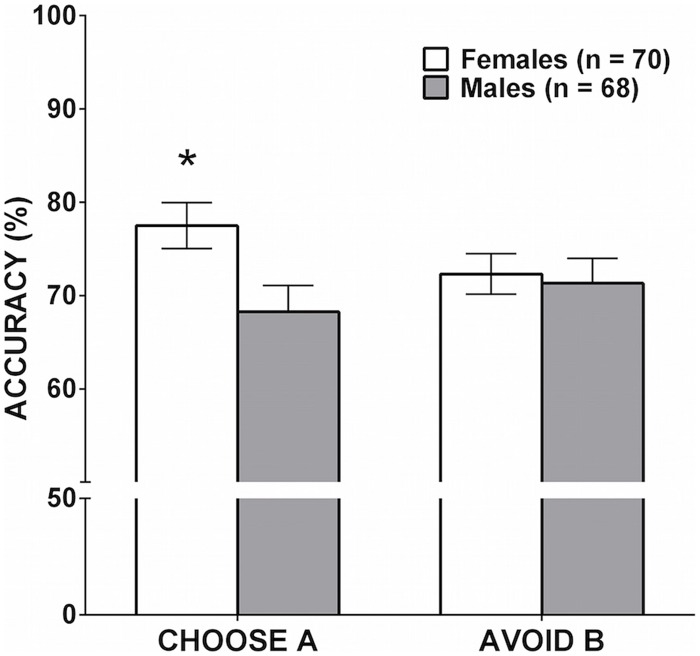
**Mean accuracy during the testing phase on the PST as a function of sex.** Error bars represent SEM. There was no significant sex difference in learning from negative feedback (avoiding B), but the difference between males and females in learning from positive feedback (choosing A) was significant (^∗^*p* = 0.015).

To test the hypothesis of a sex difference in learning from positive or negative feedback, the number of trials in which participants successfully chose A or successfully avoided B during the test phase were entered as dependent variables into a MANOVA with sex (male, female) as a between-subjects factor. There was one outlier on the Choose A accuracy measure who scored greater than three SD below the mean. The MANOVA accordingly was run without the outlier. Sex was significant in the overall MANOVA [*F*(2,135) = 3.21, *p* = 0.044, $\eta_{p}^{2}$ = 0.045]. As shown in **Figure 3**, females were significantly more accurate in choosing A during the test phase (learning from positive feedback) compared to males, *F*(1,136) = 6.09, *p* = 0.015, $\eta_{p}^{2}$ = 0.043. There was no significant difference between males and females in successfully avoiding B (learning from negative feedback), *F*(1,136) = 0.08, *p* = 0.772, $\eta_{p}^{2}$ = 0.001.

#### Arrows Task

The Arrows data were analyzed using mixed-design MANOVA with trial type (congruent, incongruent) as a within-subjects factor and sex as a between-subjects factor. The dependent variables were accuracy and RT. In the overall MANOVA, both sex [*F*(2,133) = 8.22, *p* < 0.001, $\eta_{p}^{2}$ = 0.110] and trial type [*F*(2,133) = 74.57, *p* < 0.001, $\eta_{p}^{2}$ = 0.529] were significant. The interaction of sex and trial type approached significance [*F*(2,133) = 2.92, *p* = 0.057, $\eta_{p}^{2}$ = 0.042].

For accuracy (data not shown), there was a significant main effect of trial type [*F*(1,134) = 48.91, *p* < 0.001, $\eta_{p}^{2}$ = 0.267] such that accuracy was higher on congruent than incongruent trials, as expected. This confirms the classic Simon Effect. There was no significant main effect of sex [*F*(1,134) = 0.26, *p* = 0.612, $\eta_{p}^{2}$ = 0.002] and no significant interaction between sex and trial type, *F*(1,134) = 1.41, *p* = 0.237, $\eta_{p}^{2}$ = 0.010.

**FIGURE 4:**
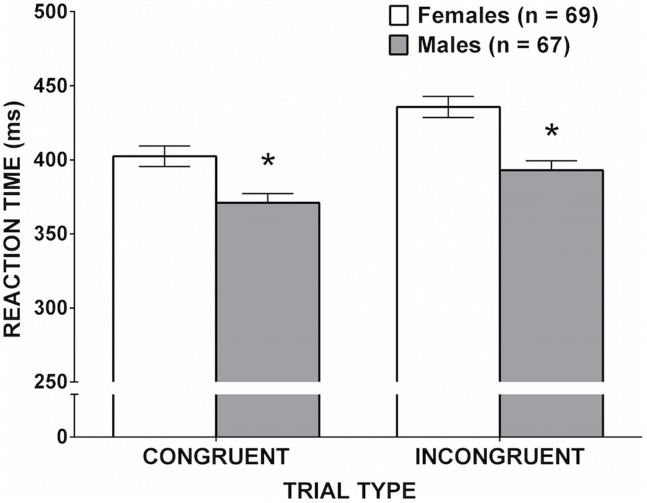
**Mean reaction time on the Arrows task as a function of sex.** Error bars represent SEM. Males were faster than females in both conditions, but the sex difference was significantly larger for incongruent than for congruent trials. (^∗^*p* = 0.019).

The RT data are shown in **Figure 4**. A significant Simon Effect was confirmed, whereby there was a main effect of trial type [*F*(1,134) = 135.53, *p* < 0.001, $\eta_{p}^{2}$ = 0.503]. Reaction times were shorter on congruent than incongruent trials. The main effect of sex was significant [*F*(1,134) = 16.39, *p* < 0.001, $\eta_{p}^{2}$ = 0.109]; males made faster responses than females. Importantly, the interaction between sex and trial type was also significant, *F*(1,134) = 5.60, *p* = 0.019, $\eta_{p}^{2}$ = 0.040, indicating that the magnitude of the male RT advantage was larger on incongruent trials, where the inhibition of a prepotent response was required.

#### Stop-Signal Task

The Stop-Signal data were analyzed using MANOVA with sex as a between-subjects factor. As described in Section “Stop-Signal Task (Cambridge Neuropsychological Test Automated Battery; CANTABeclipse, Cambridge Cognition Ltd., UK),” the task had four dependent variables. The SSRT, which is the RT to successfully inhibit a pre-programmed motor response, was the dependent variable of interest for testing the theoretical hypothesis of a male advantage. However, to properly interpret the data, the median reaction time on go trials, the proportion of successful stops, and SSD, also were analyzed (see **Table 1**). In the overall MANOVA, the main effect of sex was not significant [*F*(4,124) = 1.33, *p* = 0.264, $\eta_{p}^{2}$ = 0.041]. Therefore, follow-up univariate tests were not performed.

**Table 1:** Performance measures on the Stop-Signal Task for males and females.

	Males	Females
Measure	(*n* = 59)	(*n* = 70)
Median go reaction time	364.08 (77.33)	399.43 (102.47)
Proportion of successful stops	0.500 (0.06)	0.504 (0.07)
SSD	192.89 (95.35)	233.11 (111.65)
SSRT	171.20 (41.55)	166.41 (41.26)

### Control Tasks

#### Verbal Meaning Test

As expected, ANOVA showed no significant sex difference on the Verbal Meaning Test [Sex: *F*(1,136) = 0.49, *p* = 0.484, $\eta_{p}^{2}$ = 0.004]. Males (*M* = 28.93, SD = 9.68) and females (*M* = 27.81, SD = 8.94) achieved a similar mean score.

#### PANAS

Analysis of the mood scores unexpectedly revealed a significant main effect of sex on the PANAS Positive Affect score [Sex: *F*(1,136) = 4.07, *p* = 0.046, $\eta_{p}^{2}$ = 0.029]. Females had lower Positive Affect than did males (see **Table 2**). Negative Affect did not differ significantly [Sex: *F*(1,136) = 0.44, *p* = 0.508, $\eta_{p}^{2}$ = 0.003]. Positive Affect was not a significant covariate if entered into any of the above reported analyses (data not shown) suggesting that differences in Positive Affect did not play a significant role in the present findings.

**Table 2:** Mean (SD) scores of males and females on the PANAS.

	Males (*n* = 68)	Females (*n* = 69)
Positive affect	30.10 (6.03)	27.81 (7.23)^∗^
Negative affect	14.10 (4.29)	14.59 (4.38)

*^∗^*p* < 0.05.*

## Discussion

A male advantage has been reported on certain reinforcement learning tasks such as the IGT and reversal learning (e.g., Overman et al., 1996; Reavis and Overman, 2001; Weller et al., 2009). These tasks are functionally complex and involve many component processes that could be the source of the male advantage. A critical determinant of performance on both the IGT and reversal learning is the ability to flexibly alter behavior in response to valenced feedback. The need for inhibitory control and the processing of reward- and/or punishment-related cues are two functional task components that might be significant vis-a-vis the sex difference. The objective of the current study was to begin to identify task component(s) that are important for eliciting the sex difference in reinforcement learning.

Previous research has found a male advantage in young children (Overman et al., 1996) and in adults (Evans and Hampson, 2015) on certain reversal learning tasks. One hypothesis, tested here, is the possibility that a sex difference exists in learning from positive or negative feedback. Data from the PST supported this hypothesis where, in the absence of any reversals, females showed significantly higher accuracy than males in choosing the stimulus that had been rewarded during the learning phase. Stronger learning from positive reinforcement may be an asset on the PST yet lead to poorer performance on the probabilistic reversal task or IGT where, given the structure of those tasks, responsiveness to reward can lead to less advantageous patterns of responding.

A sex difference in the response to valenced feedback would be consistent with several previous observations and conjectures. A sex difference on the IGT has been documented (e.g., Overman, 2004; Goudriaan et al., 2007; Weller et al., 2009; van den Bos et al., 2013), whereby males select more cards from the advantageous decks during acquisition than do females. The sex difference appears to be driven largely by a consistent difference in the preference shown for a deck that has frequent, large rewards, and infrequent punishments (i.e., females select more cards from this particular deck even though, objectively, the deck leads to reduced winnings over the long term; e.g., Overman, 2004; Overman et al., 2006, 2011; van den Bos et al., 2013). This finding has led to speculation that a difference might exist in how the sexes use reward and punishment to guide IGT performance (Overman, 2004; Overman et al., 2006). It has been suggested that females rely more than males on immediate reward and punishment cues, and do so for a longer period of time, whereas males more rapidly adopt a perspective focused on long-term payoffs allowing them to select the advantageous response options on the IGT (Overman et al., 2011; van den Bos et al., 2013). In further work using a different gambling task, females showed a larger response to reward, but not punishment, compared to males as indexed by an electrophysiological measure (feedback-related negativity; Santesso et al., 2011). On the other hand, Moeller and Robinson (2010) discovered that females slowed their responses during a categorization task in response to error feedback to a larger degree than males and suggested this reflects a sex difference in punishment sensitivity, although it should be noted that responses to positive feedback were not measured. Thus data from previous work and tentatively the current study support the general hypothesis that a sex difference may exist in the processing of, or sensitivity to, valenced feedback.

A sex difference in responding to valenced feedback may not be the only task component contributing to a male advantage on the IGT or reversal learning. Such tasks require inhibitory control processes. In the current study, a male advantage was found on Arrows, but not on the Stop-Signal Task. These two tasks measure different aspects of response inhibition, and in that sense the present dissociation may be theoretically informative. Arrows assesses interference inhibition, whereas the Stop-Signal Task assesses action cancelation. It is possible that a sex difference could exist in one form of inhibitory control, but not the other. Although dedicated studies of sex differences do not exist in the current literature, the dissociation seen in the present work is supported by the limited data available. A male advantage has been reported during a task that involved inhibiting responses to obvious stimuli (numbers shown counting forward) in favor of less obvious stimuli (numbers shown counting backward) (Halari and Kumari, 2005; Halari et al., 2005) and a few studies using other interference inhibition tasks (i.e., the Flanker task) have also found that males are faster and make fewer errors than do females (Stoet, 2010; Clayson et al., 2011). An fMRI study by Christakou et al. (2009) found a sex difference in the pattern of brain activation elicited during a Simon task. Also in agreement with the current findings, past studies typically found no sex differences on measures of inhibitory control that involve the cancelation of a prepotent action, including the Stop-Signal task (Williams et al., 1999; Li et al., 2006, 2009; Cross et al., 2011; but see Thakkar et al., 2014). Thus, it may be the case that a male advantage perhaps exists on inhibitory control tasks involving interference, but not on inhibitory tasks that involve cancelation of an action.

If, in fact, males do have enhanced inhibitory control under interference and females focus more on reward during task performance, then it may help to explain why males perform better than females on the IGT and on reversal learning tasks where responses must be learned and re-learned through the provision of both positive and negative feedback. With respect to reversal, previous studies finding a male advantage (Clark and Goldman-Rakic, 1989; Overman et al., 1996; Evans and Hampson, 2015) have employed tasks that utilize positive and negative feedback to learn which stimuli are correct. However, when a reversal occurs, it is negative information (punishment or omission of reward) that is most relevant for learning the new task contingencies. Thus, in such studies, males may have the advantage if they more readily inhibit responses to previously rewarded stimuli in the face of interference when contingencies suddenly change, whereas females, if they are more focused on reward, may take longer to learn the new task contingencies when they are signaled by negative feedback. This explanation is in line with a previous suggestion that individuals who have stronger responses to reward or who are more sensitive to the opportunity to gain reward are at the same time worse at response inhibition when pre-potent responses are involved (Weinstein and Dannon, 2015).

Two forebrain circuits that help to regulate decision-making functions may be relevant to the behavioral sex differences observed in the present study. The affective loop involving the OFC, amygdala, and ventral striatum is proposed to be responsible for responding to valenced stimuli and adjusting behavior based on changing contingencies, whereas the cognitive loop comprising the dorsolateral PFC, anterior cingulate cortex, and the dorsal striatum is responsible for suppression of undue responding to stimuli that have been deemed irrelevant or distracting (van den Bos et al., 2013). Sex differences in brain activation observed during IGT performance support this hypothesis; Bolla et al. (2004) found greater activation in men than women of the lateral OFC and dorsolateral PFC during IGT performance, whereas women activated the medial OFC to a greater extent than men. Indeed, recent neuroimaging work using a reversal task suggests the lateral OFC is involved in modulating the weights of stimulus–response mappings to override a routine response, whereas activation in the medial OFC is correlated with processing and evaluation of rewarding, positive feedback (Hampshire et al., 2012). Perhaps sex differences in OFC activation are also relevant to the behavioral sex differences observed in the current study. Future work should investigate sex differences in brain activation during the processing of valenced feedback and during interference inhibition.

One way for sex differences in reward or punishment-based processing and inhibitory control to be mediated is via sex differences in neurochemistry. Both serotonin and dopamine have been implicated in the processing of valenced feedback (Rogers et al., 2003; Finger et al., 2007; Cools et al., 2008, 2009) and in inhibitory control in human studies (Crockett et al., 2009). For example, individuals with low dopamine synthesis in the striatum were found to be better at reversals based on punishment, whereas individuals with high dopamine synthesis were better at reversals based on reward (Cools et al., 2009). A growing body of evidence supports the idea that there are sex differences in both the serotonergic and dopaminergic systems (see Cosgrove et al., 2007 for a review), possibly attributable to differences in gonadal steroids. Given that past work has shown performance on the PST is sensitive to dopamine manipulation (Frank et al., 2004), future research should examine whether the sex difference on the PST is also influenced by changes in the dopaminergic system.

Another direction for future work will be to explicitly examine the role of hormones in reward/punishment processing and inhibitory control. Circulating testosterone levels predict performance on the IGT in humans (Reavis and Overman, 2001; van Honk et al., 2004; Stanton et al., 2011; Evans and Hampson, 2014), but organizational effects might also contribute. Clark and Goldman-Rakic (1989) found the male advantage in object reversal learning in infant monkeys could be eliminated by testosterone propionate treatment in females. The menstrual cycle might also prove to be important. We did not control for phase of the menstrual cycle in females as this is a complex undertaking requiring day count methods along with the assessment of hormone levels. However, future research should account for the menstrual cycle given that sex differences were, in fact, identified in the current study, providing a justification for more in-depth future investigations.

It should be noted that the present study examined sex differences in valenced feedback processing and interference inhibition, but did not examine the “pure” reversal element intrinsic to previously used reinforcement learning tasks, owing to the inherent difficulty of manipulating reversal in the absence of providing feedback. Our data thus do not rule out the possibility of a sex difference in the ability to flexibly alter a previously learned stimulus–feedback association, independent of reward and punishment contingencies. Finally, it is unclear whether there might be a difference in reward value between receiving points or monetary reward (in the IGT) and receiving correct/incorrect feedback (in the PST). The latter is arguably a better simulation of real-world decision-making, which typically does not elicit any direct monetary reward, but future work should examine the sex difference with respect to different types of reinforcers.

As also discussed by van den Bos et al. (2013), an important unresolved question is why, theoretically, a sex difference would exist in valenced feedback processing and interference-related inhibitory control. One possibility is that the observed differences between males and females in these cognitive functions are epiphenomenal, serving no adaptive function. A more satisfying explanation is that variations in sex steroids and their effects on cognitive functions have been selected because they increase adaptive behaviors and some theorists speculate that changes in the reactivity of the reward system via the modulatory influence of sex steroids play a role in facilitating procreation through changes in receptivity or desire (Caldú and Dreher, 2009). Future work should take on the goal of answering the question of why sex differences might exist in reward processing and interference inhibition.

The current study provides preliminary support for the hypothesis that females are more focused on positive feedback during reinforcement learning than males and that males are more quickly reactive than females in the face of interference. Replication of the current findings in different populations, on different tasks, and conceivably in different endocrine states, is important given the evolutionary factors that may influence how the sexes respond to reward and interference. Future research should continue to tease apart the factors that contribute to performance on complex reinforcement learning tasks in an effort to better understand the sex differences that have been demonstrated and the biological or contextual mechanisms that are responsible for those differences.

## Author Contributions

Both KE and EH had substantial contributions to the conception and design of the work; the acquisition, analysis, and interpretation of data; drafting the manuscript and revising it critically for intellectual content; final approval of the version to be published; and agree to be accountable for all aspects of the work in ensuring that questions related to the accuracy or integrity of any part of the work are appropriately investigated and resolved.

## Conflict of Interest Statement

The authors declare that the research was conducted in the absence of any commercial or financial relationships that could be construed as a potential conflict of interest.

## Abbreviations

IGT: Iowa Gambling Task
OFC: orbitofrontal cortex
PANAS: Positive and Negative Affect Schedule
PFC: prefrontal cortex
PST: probabilistic selection task
SSD: stop-signal delay
SSRT: stop-signal reaction time
VMPFC/OFC: ventromedial prefrontal cortex/orbitofrontal cortex
